# NF-κB and pSTAT3 synergistically drive G6PD overexpression and facilitate sensitivity to G6PD inhibition in ccRCC

**DOI:** 10.1186/s12935-020-01576-2

**Published:** 2020-10-06

**Authors:** Qiao Zhang, Zhe Yang, Yueli Ni, Honggang Bai, Qiaoqiao Han, Zihan Yi, Xiaojia Yi, Yannick Luther Agbana, Yingmin Kuang, Yuechun Zhu

**Affiliations:** 1 Department of Biochemistry and Molecular Biology, School of Basic Medical Sciences, Kunming Medical University, No. 1168 Yuhua Road, Chenggong District, Kunming, 650500 Yunnan China; 2 Department of Pathology, The First Affiliated Hospital of Kunming Medical University, No. 295 Xichang Road, Wuhua District, Kunming, 650032 Yunnan China; 3 Department of Clinical Laboratory, The Second Hospital of Jingzhou, No. 241 Jiangjin Road, Shashi District, Jingzhou, 434000 Hubei China; 4 Department of Medical Oncology, The Third Affiliated Hospital of Kunming Medical University (Tumor Hospital of Yunnans Province), No. 519 Kunzhou Road, Xishan District, Kunming, 650118 Yunnan China; 5 Department of Pathology, The Second Affiliated Hospital of Kunming Medical University, No. 374 Dianmian Road, Wuhua District, Kunming, 650101 Yunnan China; 6 Department of Organ Transplantation, The First Affiliated Hospital of Kunming Medical University, No. 295 Xichang Road, Wuhua District, Kunming, 650032 Yunnan China

**Keywords:** G6PD, ccRCC, ROS, NF-κB, pSTAT3, Proliferation inhibition

## Abstract

**Background:**

Glucose 6-phosphate dehydrogenase (G6PD) serves key roles in cancer cell metabolic reprogramming, and has been reported to be involved in certain carcinogenesis. Previous results from our laboratory demonstrated that overexpressed G6PD was a potential prognostic biomarker in clear cell renal cell carcinoma (ccRCC), the most common subtype of kidney cancer. G6PD could stimulate ccRCC growth and invasion through facilitating reactive oxygen species (ROS)-phosphorylated signal transducer and activator of transcription 3 (pSTAT3) activation and ROS-MAPK-MMP2 axis pathway, respectively. However, the reasons for ectopic G6PD overexpression and the proliferation repressive effect of G6PD inhibition in ccRCC are still unclear.

**Methods:**

The impact of ROS accumulation on NF-κB signaling pathway and G6PD expression was determined by real-time RT-PCR and Western blot in ccRCC cells following treatment with ROS stimulator or scavenger. The regulatory function of NF-κB signaling pathway in G6PD transcription was analyzed by real-time RT-PCR, Western blot, luciferase and ChIP assay in ccRCC cells following treatment with NF-κB signaling activator/inhibitor or lentivirus infection. ChIP and Co-IP assay was performed to demonstrate protein-DNA and protein–protein interaction of NF-κB and pSTAT3, respectively. MTS assay, human tissue detection and xenograft model were conducted to characterize the association between NF-κB, pSTAT3, G6PD expression level and proliferation functions.

**Results:**

ROS-stimulated NF-κB and pSTAT3 signaling over-activation could activate each other, and exhibit cross-talks in G6PD aberrant transcriptional regulation. The underlying mechanism was that NF-κB signaling pathway facilitated G6PD transcription via direct DNA–protein interaction with p65 instead of p50. p65 and pSTAT3 formed a p65/pSTAT3 complex, occupied the pSTAT3-binding site on G6PD promoter, and contributed to ccRCC proliferation following facilitated G6PD overexpression. G6PD, pSTAT3, and p65 were highly expressed and positively correlated with each other in ccRCC tissues, confirming that NF-κB and pSTAT3 synergistically promote G6PD overexpression. Moreover, G6PD inhibitor exhibited tumor-suppressor activities in ccRCC and attenuated the growth of ccRCC cells both in vitro and in vivo.

**Conclusion:**

ROS-stimulated aberrations of NF-κB and pSTAT3 signaling pathway synergistically drive G6PD transcription through forming a p65/pSTAT3 complex. Moreover, G6PD activity inhibition may be a promising therapeutic strategy for ccRCC treatment.

## Background

Clear cell renal cell carcinoma (ccRCC) is the most prevalent subtype of kidney malignant tumor, which constitutes about 80%–90% of renal cell carcinoma (RCC). Despite the diagnosis and treatment of ccRCC have improved obviously in recent years [1, 2], most ccRCC patients still confront difficulties in early diagnosis, drug resistance, high metastasis rate (~ 34%), and short median survival time (~ 13 months) [1, 3]. Meanwhile, new cases and the number of deaths due to ccRCC are approximately 400,000 and 139,000, respectively, globally per year [4]. These situations emphasize the importance of studies on ccRCC tumorigenesis. However, the fundamental theory of ccRCC is still unclear, especially the critical molecular mechanisms underlying ccRCC initiation and development [5, 6]. Therefore, identifying novel molecular mechanisms involved in ccRCC tumorigenesis and providing evidence for effective treatment are the pivotal points for ccRCC investigation [6].

In recent years, increasing studies had focused on the metabolism disorder of human cancers [7–9], and ccRCC was generally regarded as a metabolic disease in this field [10–13]. It was reported that ccRCC had increased antioxidant capacity and carbon metabolic reprogramming [14]; tumor progression and metastasis are closely related to enhanced glutathione, cysteine/methionine and tryptophan metabolic pathways [14, 15]. These reports highlighted the importance of the ccRCC metabolic disorder in tumorigenesis. However, the important drivers of the aforementioned metabolic reprogramming, the molecular regulatory mechanisms that joint the malignant phenotype and metabolism disorder, and the maintenance of the overall metabolic homeostasis of tumor cells remain to be clarified [14, 15].

Previous studies conducted in our laboratory showed that glucose 6-phosphate dehydrogenase (G6PD), which is the first and rate-limiting enzyme of the pentose phosphate pathway (PPP) and plays critical roles in the generation of nucleotide precursors, lipid synthesis, redox balance, and maintenance of the whole cell metabolic homeostasis [16, 17], is highly expressed in ccRCC cells and predicts the poor overall survival of patients with ccRCC, indicating that G6PD exerted crucial functions in ccRCC metabolic reprogramming and tumorigenesis [18]. A further investigation descripted that the overexpressed G6PD positively regulated reactive oxygen species (ROS) generation by facilitating NADPH oxidase 4 activity, increased phosphorylated signal transducer and activator of transcription 3 (pSTAT3) activation and enhanced RCC development [19]. Moreover, G6PD could stimulate ccRCC invasion through facilitating ROS-MAPK-MMP2 axis pathway [20]. The aforementioned results suggested that how G6PD regulated the downstream target, influenced ROS production, and promoted RCC growth and invasion had partially been revealed, but why G6PD was aberrantly overexpressed during ccRCC tumorigenesis was still not completely known.

It has been reported that ROS dysregulation plays crucial impact on abnormal intracellular signal transduction [21, 22]. ROS could stimulate continuous activation of pSTAT3, NF-κB and MAPK signals [19, 23–25], and promote the occurrence and development of various types of cancer, including RCC, shin cancer and lung cancer [19, 26, 27]. NF-κB signaling over-activation has been described to contribute to RCC cell migration and invasion [28], and the high expression level of pSTAT3 S727 is expected to be an independent prognostic molecule for ccRCC patients [29, 30]. Moreover, the interactions between NF-κB and STAT3 signaling pathways were discovered in various physiological and pathological processes, such as B-cell activation [31], cancer cell starvation [32, 33], and mitochondrial fusion [34, 35]. These two pathways were rapidly activated in response to various stimuli, including cytokines, ROS, and other stresses [36]. Additionally, it was reported that a series of tumorigenesis-related genes could be regulated by pSTAT3 and NF-κB, either synergistically or individually [33], and STAT3/NF-κB signaling targeting could sensitize triple-negative breast cancer cells to cisplatin [37]. The interactions between ROS, pSTAT3, and G6PD in ccRCC have been reported previously by our laboratory [19]. We found that G6PD could stimulated ccRCC growth through facilitating ROS production and pSTAT3 signaling activities, and pSTAT3 showed a positive feedback regulation of G6PD transcription [19]. These evidences promote us to hypothesize that there may be a correlation between not only ROS and pSTAT3, but also NF-κB signaling pathway and G6PD overexpression in ccRCC. However, the cooperation between these factors, and the underlying mechanism of how they exert the role in ccRCC tumorigenesis have not yet been clarified.

Numbers studies suggested that the interaction work mode of NF-κB and STAT3 was commonly existed in certain conditions, especially in cancer cells [32–36]. It has been reported that NF-κB and STAT3 cooperate to promote the development and progression of gastric, colon, and liver cancers, which contribute to explain the molecular pathogenesis of cancers, and offer opportunities for the design of new therapeutic interventions [36]. Interactions and forms of crosstalk between NF-κB and STAT3 include physical interaction between the two, cooperation of these factors at gene promoters, the NF-κB dependent expression of STAT3 inhibitors and the participation of STAT3 in the regulation of NF-κB activation [33, 36]. In several human tumors, NF-κB and STAT3 are activated and interact. STAT3 and NF-κB exist as identical nuclear complexes and cooperatively induce various survival factors [32]. All these evidences indicated that forming a complex might be the usual functional model of NF-κB and STAT3. However, the components of the STAT3/NF-κB complex are diversity and complexity in different biological phenomena, including unphosphorylated STAT3/NF-κB subunits [32], tyrosine-phosphorylated STAT3/p65/p50 [33], acetylated STAT3/p65 [34], and others [36]. However, what is the interaction model between NF-κB and STAT3 signaling pathways, whether the STAT3/NF-κB complex exists in ccRCC, its constituents, or where the binding site of the complex is located are unknown.

The present study explored the reasons for the high expression of G6PD in ccRCC. To do so, a reciprocal effect between ROS, NF-κB/STAT3 signaling pathway and G6PD aberrant overexpression in ccRCC was consolidated. Both in vitro and in vivo investigations were conducted to unravel the molecular mechanism underlying ROS-mediated NF-κB/STAT3 signaling pathway over-activation and ectopic G6PD transcription. Moreover, to evaluate the potential role of G6PD as a therapeutic target for ccRCC treatment, the effects of G6PD inhibition on ccRCC proliferation were assessed both in vitro and in vivo.

## Methods

### Cell culture, cell treatment, and cell proliferation assays

The most highly cited cell lines that are commonly used for ccRCC researches, including ACHN (ATCC, CRL-1611™), 786-O (ATCC, CRL-1932™) and Caki-1 (ATCC, HTB-46™) [38, 39] were obtained from Chinese Academy of Sciences, Kunming Institute of Zoology (Kunming, China) and cultured in RPMI-1640 (11875-085, Gibco, Grand Island, NY, USA) containing 10% fetal bovine serum (FBS, 16140071, Thermo Fisher Scientific, Waltham, MA, USA) in a 37˚C humidified incubator (5% CO_2_).

H_2_O_2_ was purchased from Santa Cruz (sc-203336, CA, USA) and saved at 4 °C. N-acetyl-L-cysteine (NAC, A7250, Sigma, Louis, MO, USA), 6-aminonicotinamide (6-AN, A68203-1G, Sigma), STATTIC (sc-202818, Santa Cruz), and BAY11-7082 (S2913, Selleck, Shanghai, China) were dissolved in 100% DMSO to prepare a 600 mM, 250 mM, 40 mM, and 10 mM stock, respectively, and stored at − 20 °C. Recombinant human IL-6 (206IL, R&D Systems, MN, USA) and TNFα (300-01A, Peprotech, Rocky Hill, USA) were dissolved in 0.1% BSA to prepare a 10 μg/mL and 50 µg/mL stock, respectively, and stored at − 20 °C. p65-knocked down RNAi lentivirus (target sequence: CGGATTGAGGAGAAACGTAAA), p50-knocked down RNAi lentivirus (target sequence: CCTTTCCTCTACTATCCTGAA) and negative control were purchased from Genechem (Shanghai, China). An MTS cell proliferation assay kit purchased from Promega (CTB169, Beijing, China) was used for cell viability analysis according to the manufacturer′s protocol.

### Real-time RT-PCR

Total RNA was isolated and real-time RT-PCR amplifications were performed according to previous reports [19, 40] using the following primers: G6PD: F: 5′-TGAGCCAGATAGGCTGGAA-3′, R: 5′-TAACGCAGGCGATGTTGTC-3′; U6: F: 5′- CTCGCTTCGGCAGCACA -3′, R: 5′- AACGCTTCACGAATTTGCGT -3′; CyclinD1: F: 5′-GCGTACCCTGACACCCCTCTC-3′, R: 5′-CTCCTCTTCGCCTGATCC-3′; CDK4: F: 5′-ACAGTTCGTGAGGTGGCTTTAC-3′, R: 5′-GTCCTTAGGTCCTGGTCTACATG-3′; STAT3: F: 5′-AGAAGGAGGCGTCACTTTCA-3′, R: 5′-TTTCCGAATGCCTCCTCCTT-3′.

### Western blot analysis

Cell lysates were prepared and Western blot analysis was performed as previously described [19, 40]. Following antibodies were used: anti-G6PD antibody (ab133525, Abcam, Cambridge, U.K.); anti-STAT3 antibody (4904, Cell Signaling Technology), anti-phospho-STAT3 antibody (Ser 727) (ab30647), anti-CyclinD1 antibody (ab16663, Abcam), Anti-CDK4 antibody (ab108357), Anti-p105/50 antibody (3035, Cell Signaling Technology), anti-p65 antibody (ab32536), anti-pIκBα (Ser32 + Ser36) antibody (AF2002, Affinity, USA), anti-IκBα antibody (AF5002, Affinity, USA), anti-β-actin antibody (4967, Cell Signaling Technology), and anti-GAPDH antibody (2118, Cell Signaling Technology).

### Luciferase report analysis

293 T cells were seeded in a 24-well plate, and constructs of pGL3-BASIC-luc-G6PD vector (wild typeor NF-κB-binding site deletion) were transfected using Lipofectamine 2000 Transfection Reagent (11,668,019, Invitrogen) when the cells reached 60% confluence. After TNFα or BAY11-7082 stimulation for 24 h, the luciferase activity was measured using a luciferase report assay kit (T002, Vigorous Biotechnology, Beijing, China). Data were evaluated following manufacturer′s protocols.

### Chromatin Immunoprecipitation (ChIP) assays

ChIP experiments were performed as described earlier [19, 40] using the following primers covering a 101-bp potential region of the NF-κB-binding site on the G6PD promoter: F: 5′-ACGAGCAAACAGGCATATGA-3′, R: 5′-CCAAACTTGACTGCGCTCTAT-3′, or a primer covering a 103-bp potential region of the pSTAT3-binding site on the G6PD promoter: F: 5′-AACGTCCGGGGGAAGTTTC-3′, R: 5′-TTCTCACGTCTGACGGACTCT-3′. The following antibodies were used: anti-p105/50 antibody (3035, Cell Signaling Technology), anti-p65 antibody (ab32536), anti-STAT3 antibody (4904, Cell Signaling Technology), and anti-phospho-STAT3 antibody (Ser 727) (ab30647).

### Co-IP assays

The cell lysates were prepared using RIPA lysis buffer (P0013D, Beyotime, Shanghai, China) containing protease inhibitor cocktail (469313200, Roche Basle, Switzerland). Immunoprecipitation and Western blot analysis were carried out as described earlier using anti-phospho-STAT3 antibody (Ser 727) (ab30647, Abcam), anti-p65 antibody (ab32536, Abcam), or the control anti-immunoglobulin G antibody (X0936, DAKO A/S) [40].

### Immunohistochemistry (IHC)

All pathological specimens were collected from the department of pathology of the First Affiliated Hospital of Kunming Medical University, which was approved by the Research Ethics Committee of Kunming Medical University. Anti-G6PD antibody (ab133525, Abcam), anti-phospho-STAT3 antibody (Ser 727) (ab30647, Abcam), and anti-p65 antibody (ab32536, Abcam) were used for immunohistochemical analysis performed using the 2-step plus poly-HRP anti-mouse/rabbit IgG detection system (PV-9000, ZSGB-BIO, Beijing, China). The experimental procedures were performed following the manufacturer′s protocol and previous reports [19].

### G6PD activity detection

G6PD activity was analyzed using the G6PD assay kit (GMS70013.1, Genmed, Shanghai, China) following the manufacturer′s protocol and previous report [19].

### Animal experiments

Six-week-old female BALB/c nude mice were injected subcutaneously with 1 × 10^7^ ccRCC cells suspended in 200 μL of phosphate-buffered saline in the right oxter flank. Tumor volumes were calculated as follows: length × width^2^ × 0.5. When the average of tumor volume reached 200 mm^3^, tumor-bearing nude mice were randomly divided into two groups (*n* = 5 per group) and treated intratumor with vehicle or 6-AN at a dose of 23 mg/kg every day for 2 weeks. At the indicated time point, the mice were euthanized by cervical dislocation, and the tumors were harvested. All animal experiments were approved by the Institutional Animal Care and Use Committee of Kunming Medical University.

### Statistical analysis

The differences in G6PD, pSTAT3, and p65 expression levels between ccRCC and paired adjacent normal tissues in the immunohistological analysis was assessed using the *χ*^2^ test. The correlations between G6PD, pSTAT3, and p65 expression levels were assessed using Pearson correlation analysis. The significance of the difference in tumor volumes between the groups in animal experiments was determined using Mixed ANOVA. For the other analyses, the differences between groups were determined using the Student *t* test (*n* = 2) or one-way ANOVA (*n* ≥ 3). The data were expressed as means ± standard deviation (SD) of three independent experiments. A *P* value of less than 0.05 was considered to be statistically significant.

## Results

### ROS positively regulated G6PD and NF-κB signaling pathway in ccRCC cells

**Fig. 1:**
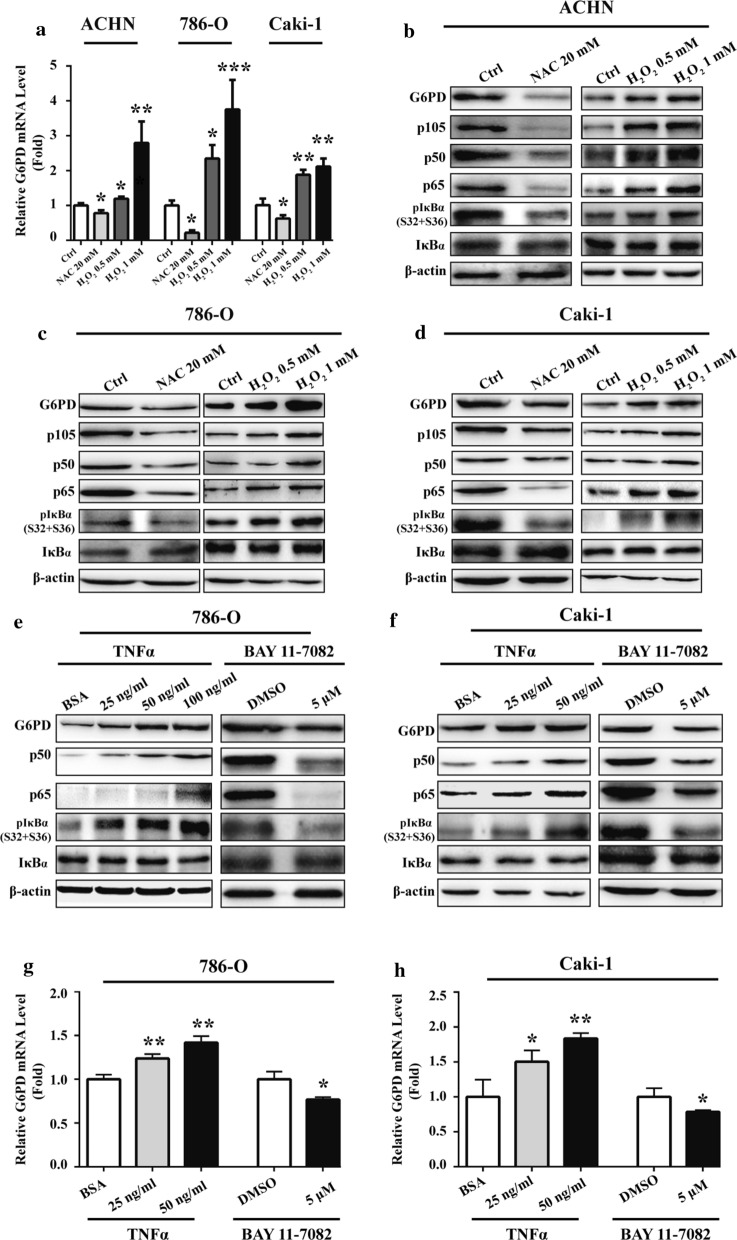
ROS positively regulated G6PD and NF-κB signaling pathway in ccRCC cells. **a**–**d** ACHN, 786-O or Caki-1 cells were treated with NAC (24 h) or H_2_O_2_ (2 h) to inhibit or induce ROS production, respectively. The changes in the expression of G6PD at the mRNA level and G6PD, p105, p50, p65, pIκBα, and IκBα at the protein level were detected using real-time RT-PCR (**a, b**) and Western blot (**c, d**) analysis, respectively. **e–h** 786-O or Caki-1 cells were treated with TNFα (24 h) or BAY11-7082 (24 h) at indicated doses to stimulate or inhibit NF-κB signaling pathway activities. The changes in the expression of G6PD, p50, p65, pIκBα, and IκBα at the protein level and G6PD at the mRNA level were detected using Western blot (**e, f**) and real-time RT-PCR (**g, h**) analysis, respectively. β-Actin was used as a loading control. Bars represent the mean ± SD from three independent experiments, each performed in triplicate. *P < 0.05, **P < 0.01, ***P < 0.001 vs each control

**Fig. 2:**
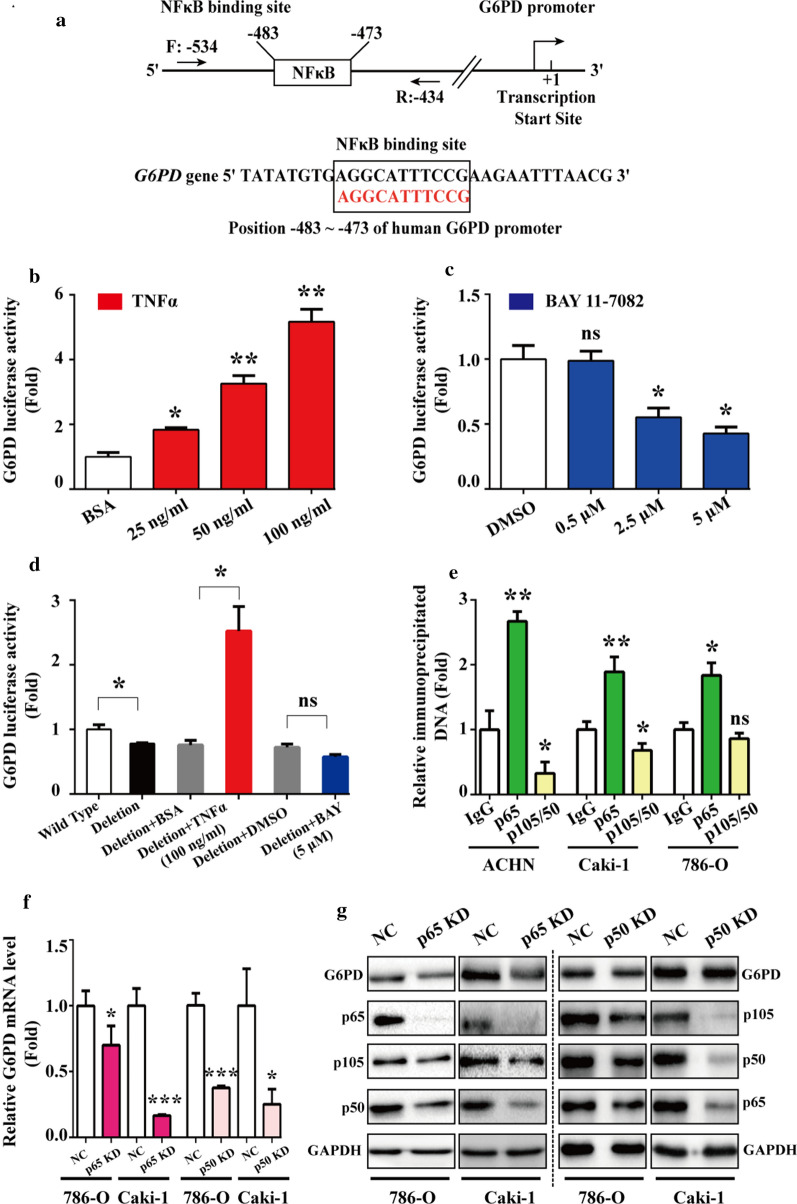
NF-κB signaling pathway facilitated G6PD expression via direct protein–DNA interaction with p65 instead of p50. **a** MatInspector software platform showed that NF-κB binding site was localized at the G6PD promoter region and the primers were designed for ChIP analysis. **b, c** The G6PD promote sequence containing the potential NF-κB binding site was cloned to a pGL3-BASIC-luc vector (named *G6PD-luc*) and conducted to luciferase report assay in 293 T cells following treatment with TNFα (24 h) (**b**) or BAY11-7082 (24 h) (**c**) at indicated doses. **d** The NF-κB-binding sequence deleted vector was used to repeat the luciferase reporter assay in 293 T cells. **e** The critical factors involved in the NF-κB signaling pathway, including p65 and p50/105, were subjected to ChIP assay in ACHN, Caki-1, and 786-O cells. **f, g** p65 or p50 was knocked down by RNAi lentivirus infection in 786-O and Caki-1 cells. The changes in the expression of G6PD at the mRNA level and G6PD, p65, p105, and p50 at the protein level were detected using real-time RT-PCR (**F**) and Western blot (**G**) analysis, respectively. GAPDH was used as a loading control. Data are expressed as mean ± SD from three independent experiments, each performed in triplicate. *P < 0.05, **P < 0.01, ***P < 0.001; ns, nonsignificant vs each control. NC, Negative control lentivirus

A pilot study revealed that G6PD-facilitated ROS accumulation could positively induce G6PD transcription in RCC [19]. To further reveal the impact of ROS on G6PD expressional regulation and discover other relevant signaling factors involved in this process, ROS production was triggered or inhibited with H_2_O_2_ or NAC in ACHN, 786-O and Caki-1 cells. The results demonstrated that G6PD mRNA expression level was significantly decreased in NAC-treated ACHN, 786-O and Caki-1 cells, whereas it was obviously increased in H_2_O_2_-stimulated cells compared with the control (Fig. 1a–d). These results were consistent with previous study using different ccRCC cell lines [19], and further confirmed the reciprocal regulatory effect between ROS and G6PD in ccRCC. Therefore, the functional factors of signaling pathways simultaneously involved in ROS and G6PD regulation were analyzed using the MatInspector software platform (will be discussed later in Fig. 2a). The results indicated that the NF-κB signaling pathway, which was demonstrated to be dysregulated and functional in the ROS-related chemotherapeutic resistance of ccRCC cells [24, 25], might intervene in the regulation of G6PD expression. To verify this hypothesis, the protein expression pattern of several important functional factors of NF-κB pathway, including p105, p50, p65, pIκBα (S32 + S36), and IκBα, which have been proved to participate in signaling transduction and closely link to ccRCC tumorigenesis [24, 25, 28, 41], was examined in NAC- or H_2_O_2_-treated ACHN, 786-O and Caki-1 cells. The results from Western blot showed that the levels of pIκBα, the most abundant inhibitory molecule of NF-κB [41], were reduced by ROS depletion, but there were increased following ROS accumulation in all these three cells. Moreover, ROS positively regulated both the NF-κB signaling activation and G6PD expression in ccRCC cells (Fig. 1b–d). Based on the previous conclusion that G6PD promoted RCC proliferation through positive feedback regulation of ROS-pSTAT3 axis [19], the present findings led to the hypothesis that ROS regulated G6PD overexpression might not only through pSTAT3 activation but also the NF-κB signaling pathway.

To further clarify the interactions between G6PD and NF-κB signaling pathway, TNFα or BAY11-7082, which could simulate or inhibit the activity of NF-κB signaling pathway, were used to treat 786-O and Caki-1 cells. Subsequently, the expression of G6PD and NF-κB pathway–related functional activators, including p50 and p65 at the protein level were evaluated. The results from western blot displayed that the protein expression levels of G6PD, p50 and p65 were increased in a dose-dependent manner in TNFα‑treated cells, whereas they were decreased in BAY11-7082‑treated cells. The phosphorylation of IκBα were increased or reduced following NF-κB signaling activation or inhibition, respectively (Fig. 1e, f). Additionally, the results from real-time RT-PCR demonstrated that the mRNA expression levels of G6PD was also increased in TNFα‑treated cells, whereas it was decreased in cells following BAY11-7082 treatment (Fig. 1g, h), which was consistent with the expression changes of G6PD at the protein level. Taken together, these findings suggested that ROS-mediated NF-κB signaling pathway may be involved in increased G6PD transcription in ccRCC.

### NF-κB signaling pathway facilitated G6PD expression via direct protein–DNA interaction with p65 instead of p50

To explore the underlying mechanism of NF-κB signaling pathway regulated G6PD expression in ccRCC, MatInspector software platform was used to analyze the potential regulatory factors that could bind to G6PD promoter. Fortunately, the binding site of NF-κB signaling–related factor was found to occupy the –483 to –473 bp of the G6PD promoter (Fig. 2a). Subsequently, a sequence containing this potential binding site along with the confirmed pSTAT3 binding site on G6PD promoter [19] was cloned to pGL3-BASIC vector (named *G6PD-luc*), and luciferase report assay was performed to determine the impact of TNFα or BAY11-7082 on the luciferase activity in 293 T cells. As presented in Fig. 2b, c, the luciferase activity of *G6PD-luc* was significantly increased by 4.2-fold following TNFα (100 ng/ml) treatment, whereas the luciferase activity was significantly decreased by about 37% following BAY11-7082 (5 μM) treatment. Additionally, repeated luciferase reporter assay using a mutant vector with the NF-κB-binding sequence deleted (*G6PD-luc-D*) displayed that the luciferase activity was significantly decreased compared with the wild type vector. Moreover, in TNFα-treated cells, the luciferase activity was increased by only 2.3-fold; however, in BAY11-7082-treated cells, the luciferase activity was decreased by no more than 21% (Fig. 2d), which demonstrated that the regulatory effect of NF-κB signaling pathway on G6PD expression was obviously attenuated without NF-κB-binding sequence. These findings indicated that NF-κB signaling pathway may promote G6PD overexpression through a direct transcriptional regulatory effect.

In order to explore how NF-κB signaling pathway contributes to G6PD transcription, oligonucleotide primers covering the potential NF-κB binding site were designed for ChIP analysis in ACHN, Caki-1, and 786-O cells by using anti-p65 or anti-p105/50 antibodies. The results demonstrated that p65 instead of p105/50 displayed a space-occupying effect (Fig. 2e), indicating that p65 might exert a direct regulatory effect on the G6PD promoter. To further evaluate the role of p65 in G6PD expression, the endogenous expression of p65 was knocked down with RNAi lentivirus in 786-O and Caki-1 cells. The results from real-time RT-PCR and Western blot demonstrated that both G6PD mRNA and protein expression levels were significantly declined compared with the negative control (Fig. 2f, g), which suggested that p65 could directly regulate G6PD transcription by occupying the binding site on the G6PD promoter. Additionally, the expression of G6PD mRNA levels was obviously decreased following p50 RNAi lentivirus transfection in 786-O and Caki-1 cells, whereas the results of Western blot analysis demonstrated that the G6PD protein expression levels were not significantly modified by p50 knocked down (Fig. 2f, g). Taken together, these findings confirmed the hypothesis that NF-κB signaling pathway facilitated G6PD expression via direct protein–DNA interaction with p65 instead of p50.

### p65 and pSTAT3 presented a synergistic effect on G6PD transcriptional regulation

**Fig. 3:**
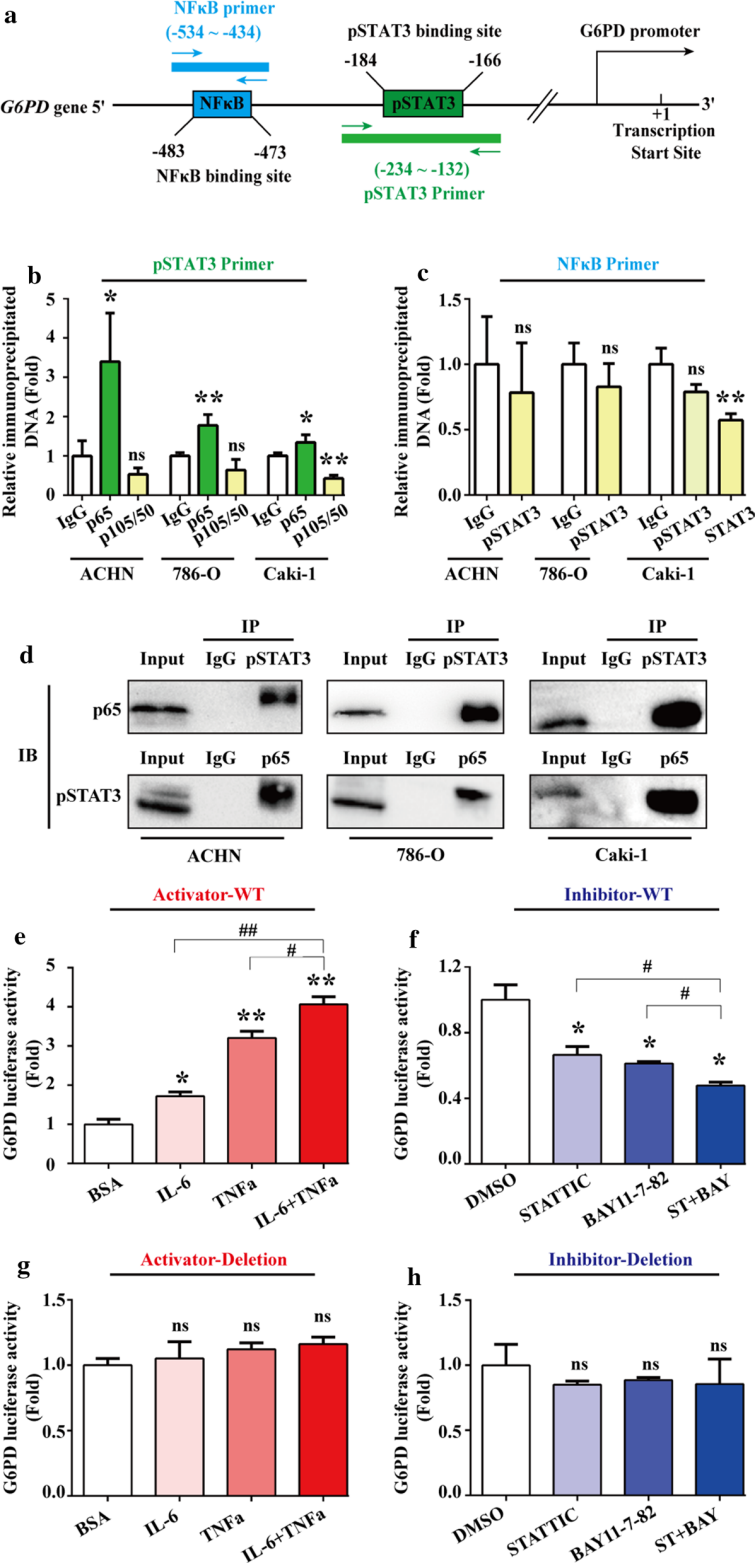
p65 and pSTAT3 presented a synergistic effect on G6PD transcriptional regulation. **a** MatInspector software platform showed that the potential NF-κB- and STAT3-binding sites localized in the G6PD promoter region were adjacent to each other. Primers covering each indicated transcriptional factor–binding region were designed. **b** ChIP assay was performed with anti-p65 or p50/105 antibodies in ACHN, 786-O, and Caki-1 cells, and the eluate was amplified by real-time RT-PCR with primers covering the pSTAT3-binding site. **c** Similar experiments were repeated with anti-pSTAT3 or STAT3 antibody in ACHN786-O, and Caki-1 cells, and primers covering the NF-κB-binding site was used. **d** The interaction between pSTAT3 and p65 was determined by Co-IP in ACHN, 786-O, and Caki-1 cells. **e–h** The luciferase activity of *G6PD-luc* WT containing the NF-κB and pSTAT3 binding sites (**e, f**) and *G6PD-luc* Deletion without both the NF-κB- and pSTAT3-binding sequence (**g, h**) were analyzed in 293 T cells following treatment with the STAT3 or NF-κB signaling activator (IL-6, 2 ng/mL or TNFα, 50 ng/mL) (**e**, **g**), or inhibitor (STATTIC, 3 μM or BAY11-7082, 2.5 μM) (**f**, **h**) independently or jointly for 24 h. Data are expressed as mean ± SD from three independent experiments, each performed in triplicate. *P < 0.05, **P < 0.01, ***P < 0.001, ^#^*P* < 0.05, ^##^*P* < 0.01; ns, nonsignificant vs each control. ST, STATTIC; BAY, BAY11-7082

In a previous study conducted in our laboratory, it was found that G6PD was regulated by pSTAT3 through directly binding to –1614 bp to –1595 bp of the G6PD promoter region [19]. Interestingly, the potential p65-binding site is just adjacent to the functionally verified pSTAT3-binding site, which is located –184 to –166 bp on the G6PD promoter calculated from the transcriptional start site (Fig. 3a), indicating that pSTAT3 and p65 might co-occupy the binding region and interact with each other to synergistically promote G6PD overexpression. To testify this hypothesis, the ChIP assay was performed in three cell lines including ACHN, 786-O and Caki-1. Firstly, cell lysates were immunoprecipitated with anti-p65 or anti-p105/50 antibody, and the eluates were amplified with primers covering the pSTAT3-binding site (Fig. 3b). Conversely, cell lysates were immunoprecipitated with anti-pSTAT3 (S727) or anti-STAT3 antibody, and the eluates were amplified with primers covering the NF-κB-binding site (Fig. 3c). The analysis revealed that p65 instead of p50 was capable of binding to the pSTAT3-binding site (Fig. 3b), but neither pSTAT3 (S727) nor STAT3 interacted with the NF-κB effective binding site (Fig. 3c). These results indicated that pSTAT3 and p65 might form a complex and bind to the pSTAT3- rather than the NF-κB-binding site. In this G6PD transcriptional regulatory model, although p65 had the potential to regulate G6PD transcription independently, it could also form a p65/pSTAT3 transcriptional complex and occupy pSTAT3 but not its own binding site while displaying a synergistic effect with pSTAT3.

To consolidate this hypothesis, the Co-IP assay was conducted in ACHN, 786-O and Caki-1 cells to investigate the protein–protein interaction between pSTAT3 and p65. The results demonstrated that the substantial interaction between pSTAT3 and p65 indeed existed in ccRCC cells (Fig. 3d), which suggested that there may be an interaction between STAT3 and NF-κB signaling pathways on G6PD transcriptional regulation. Furthermore, results from luciferase report assay demonstrated that the luciferase activity of *G6PD-luc* containing both the NF-κB and pSTAT3 binding sites was significantly increased following the treatment of STAT3 or NF-κB signaling pathways activator (Fig. 3e), whereas it was decreased following the treatment of STAT3 or NF-κB signaling pathways inhibitor (Fig. 3f). Moreover, synergistic effects could be seen in combinative stimulation with these two signaling pathways activator or inhibitors (Fig. 3e, f). However, the luciferase activities were not obviously modified following NFκB and/or pSTAT3 signaling activator/inhibitors stimulation by using a mutant vector with both the NF-κB- and pSTAT3-binding sequence deletion (Fig. 3g, h). Taken together, these findings demonstrated that STAT3 and NF-κB signaling pathways presented a cross-talk and synergistic effect on G6PD transcription. The underlying mechanism was that p65 and pSTAT3 formed a p65/pSTAT3 complex, occupied the pSTAT3-binding site of the G6PD promoter, and synergistically facilitated G6PD overexpression in ccRCC.

### NF-κB and STAT3 activated each other and facilitated ccRCC proliferation synergistically

**Fig. 4:**
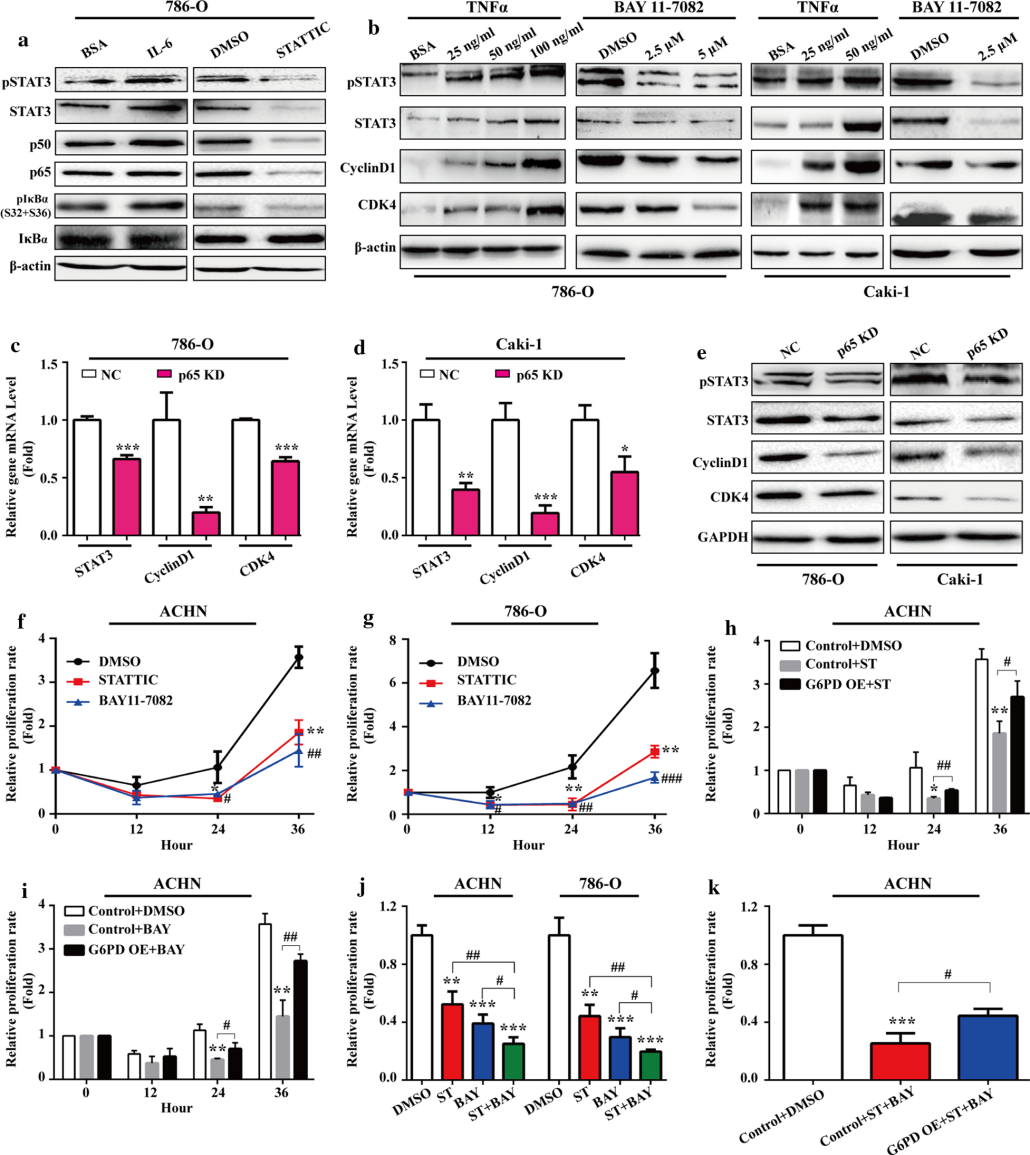
NF-κB and STAT3 activated each other and facilitated ccRCC proliferation synergistically. **a** 786-O cells were treated with pSTAT3 stimulator IL-6 (4 ng/mL) or inhibitor STATTIC (6 μM) for 24 h. The changes in the expression of pSTAT3, STAT3, p50, p65, pIκBα, and IκBα at the protein level were detected using Western blot analysis. **b** 786-O or Caki-1 cells treated with TNFα (24 h) or BAY11-7082 (24 h) at indicated doses were subject to Western blot analysis to determine the protein expression changes of pSTAT3, STAT3, CyclinD1, and CDK4. 786-O or Caki-1 cells were infected with p65 RNAi lentivirus or negative control. The changes in the expression of STAT3, CyclinD1, and CDK4 at the mRNA level, and pSTAT3, STAT3, CyclinD1, and CDK4 expression at the protein level were determined by real-time RT-PCR **c, d** and Western blot (**e**) analysis, respectively. The relative proliferation rates of ACHN (**f**) or 786-O (**g**) cells following treatment with DMSO (control), STATTIC (pSTAT3 inhibitor, 6 μM), or BAY11-7082 (NF-κB inhibitor, 5 μM) were measured by MTS assay at indicated time course. (**H-I**) The control or G6PD-overexpressing ACHN cells were treated with STATTIC (6 μM) (**h**), or BAY11-7082 (5 μM) (**i**) for 0, 12, 24, and 36 h, and the relative proliferation rate was determined by MTS assay. **j** ACHN and 786-O cells were treated with STATTIC (6 μM) or BAY11-7082 (5 μM) independently or jointly for 36 h, and the relative proliferation rate was measured by MTS assay. **k** The control or G6PD-overexpressing ACHN cells were treated with DMSO or combination of STATTIC (6 μM) and BAY11-7802 (5 μM) for 36 h, and the relative proliferation rate was determined by MTS assay. β-Actin or GAPDH was used as a loading control. Data are expressed as mean ± SD from three independent experiments, each performed in triplicate. *P < 0.05, **P < 0.01, ***P < 0.001, ^#^*P* < 0.05, ^##^*P* < 0.01, and ^###^*P* < 0.001 vs each control. NC, negative control; ST, STATTIC; BAY, BAY11-7082; G6PD OE, G6PD overexpression

The aforementioned results suggested that STAT3 and NF-κB signaling pathways over‑activation may synergistically contribute to ccRCC proliferation following G6PD overexpression. To further evaluate the reciprocal effect between these two pathways and the role of NF-κB/STAT3-mediated G6PD overexpression in ccRCC growth, 786-O cells were stimulated with the STAT3 signaling activator (IL-6) or inhibitor (STATTIC) to promote or inhibit STAT3 phosphorylation, respectively. The results from Western blot showed that the expression of p50, p65, and pIκBα were significantly increased following treatment with IL-6, whereas they were decreased following treatment with STATTIC (Fig. 4a), which suggested that the STAT3 phosphorylation status could influence the activation of NF-κB signaling pathway. Likewise, NF-κB over‑activation or inhibition could also positively impacted the STAT3 signaling pathway activation in a dose-dependent manner (Fig. 4b). These findings indicated a reciprocal regulatory effect between NF-κB and STAT3 signaling pathways in ccRCC.

It has been reported that aberrant NF-κB activation depends on CDK4/6-mediation and contributes to tumor cell progression [42]. Previous results from our laboratory demonstrated that pSTAT3 excessive activation contributed to G6PD-stimulated RCC cell proliferation via upregulated CyclinD1 expression [19]. In the present study, the role of NF-κB and STAT3 cross-talk in ccRCC growth was further explored. It was found that NF-κB positively regulated not only CyclinD1 but also CDK4 expression at the protein level (Fig. 4b), which suggested that NF-κB might synergistically cooperate with STAT3 and in part contribute to ccRCC proliferation following G6PD overexpression. To confirm this hypothesis, endogenous p65 expression was knocked down by infected 786-O and Caki-1 cells with RNAi lentivirus. The results from real-Time RT-PCR and Western blot demonstrated that the expression of STAT3, CyclinD1 and CDK4 were significantly decreased at the mRNA and protein level in p65 knocked down cells (Fig. 4c–e). These findings suggested that NF-κB and STAT3 signaling pathways could activate each other and exhibit a synergistical proliferation-promoting regulatory effect in ccRCC.

To further evaluate the role of NF-κB/STAT3-mediated G6PD overexpression in ccRCC growth, the MTS assay was conducted to determine the proliferation rate of ACHN and 786-O cells following treatment with STATTIC or BAY11-7082, respectively. As shown in Fig. 4f, g, both NF-κB and STAT3 inhibition presented a proliferation-inhibiting effect on ACHN and 786-O cells. However, whether G6PD overexpression is involved in this growth suppression effect needs to be clarified. Therefore, further MTS analyses were performed in G6PD‑overexpressing ACHN and the relevant control cells to detect the proliferation-inhibiting effect of STATTIC or BAY11-7082. As presented in Fig. 4h, i, G6PDoverexpression could reversed the impact of STAT3 and NF-κB inhibitor on ACHN cell proliferation. Furthermore, ACHN or 786-O cells were treated with STATTIC and BAY11-7082 independently or in combination. The results from MTS assay demonstrated that these two inhibitors displayed a synergistical growth suppression effect in both ACHN and 786-O cells (Fig. 4j), and the overexpression of G6PD in ACHN cells, which had the lowest G6PD activity in the three of the RCC cell lines, could rescue this tendency (Fig. 4k), indicating that G6PD overexpression is necessary for NF-κB/STAT3 promoted ccRCC proliferation. Taken together, these results suggested that NF-κB and STAT3 signaling pathways could activate each other, synergistically promote G6PD expression and contribute to ccRCC proliferation.

### G6PD, pSTAT3, and p65 were highly expressed and positively correlated with each other in ccRCC tissues

**Fig. 5:**
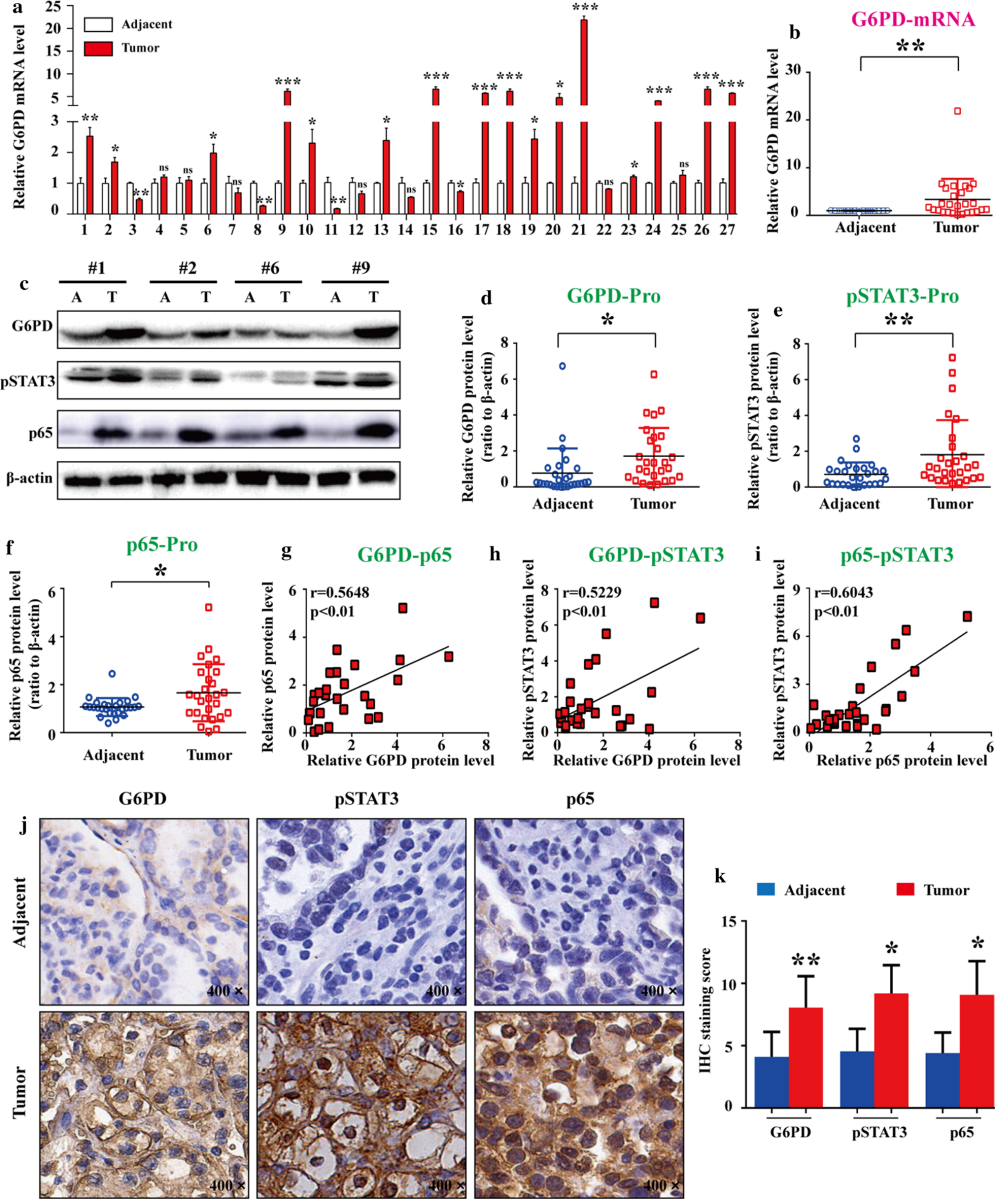
G6PD, pSTAT3, and p65 were highly expressed and positively correlated with each other in ccRCC tissues. **a, b** G6PD mRNA expression level was detected by real-time RT-PCR in 27 ccRCC tumor specimens and adjacent normal tissues. **c–f** All specimens were subjected to Western blot analysis using anti-G6PD, pSTAT3, or p65 antibodies. The representative Western blot images (**c**) and the statistics of quantitative analysis were shown (**d–f**). **g–l** The correlations between G6PD, pSTAT3, and p65 at the protein level were tested using Pearson correlation analysis. **j** Immunohistochemistry staining of G6PD, pSTAT3, and p65 in these specimens was conducted, and representative images are shown (200 ×). **k** Quantification of the staining score from **j**. Data were expressed as the means ± SD. *P < 0.05, **P < 0.01, ***P < 0.001; ns, nonsignificant vs adjacent normal tissue

To verify the aforementioned mechanistic model in vivo, G6PD expression profile was evaluated in 27 ccRCC tumor species and matched adjacent normal tissues by real-time RT-PCR. The results demonstrated that 16 of 27 (59.3%) tumor species exhibited high G6PD expression levels (Fig. 5a), and the statistical analysis revealed that the mRNA expression level of G6PD was significantly increased in ccRCC species compared with normal tissues (Fig. 5b). Subsequently, all the specimens were subjected to Western blot analysis for the protein expression level detection of G6PD, pSTAT3, and p65. The results demonstrated that the expression levels of all these factors were significantly increased in ccRCC tumor tissues compared with adjacent normal tissues (Fig. 5c–f). In addition, the results from Pearson correlation analysis demonstrated that G6PD, pSTAT3, and p65 were positively correlated with each other at the protein level (Fig. 5g–i). Moreover, immunohistochemistry staining was performed to analyze the expression of G6PD, pSTAT3, and p65 in tumor tissues and adjacent normal tissues. As shown in Fig. 5j, G6PD and pSTAT3 was mainly localized in the cytoplasm and nucleus of the ccRCC tumor cells, respectively; whereas the predominant localizations of p65 was seen within the whole ccRCC cells. Moreover, G6PD along with both pSTAT3 and p65 were displayed high expression levels in tumor tissues compared with adjacent normal tissues (Fig. 5j–k). Taken together, these findings demonstrated that the persistent activation of both NF-κB and STAT3 signals existed in ccRCC tissues, which may contribute to ccRCC tumorigenesis partially through the synergistically mediated G6PD overexpression.

### G6PD activity inhibition attenuated the growth of ccRCC cells both in vitro and in vivo

**Fig. 6:**
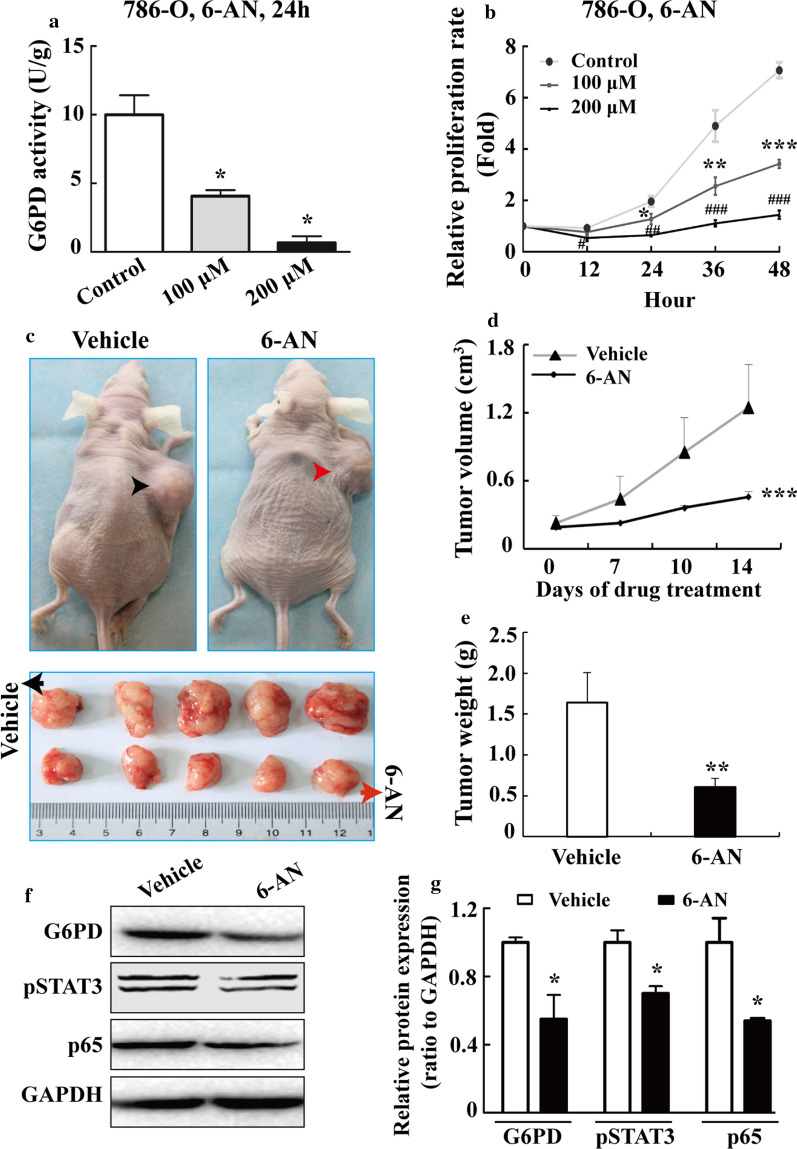
G6PD activity inhibition attenuated the growth of ccRCC cells both in vitro and in vivo. **a** 786-O cells were treated with 6-AN at indicated doses for 24 h, and reduced G6PD activities were verified using a G6PD Assay Kit. **b** After treating 786-O cells with 6-AN at indicated doses for 12, 24, 36 or 48 h, the relative proliferative rate was analyzed by MTS assay. **c** 786-O parental tumor-bearing nude mice were treated with vehicle or 6-AN for 2 weeks. Representative images of tumor-bearing mice (top panel) and tumors isolated from each group (bottom panel) are shown. **d** Tumor volumes were measured at the indicated days. **e** The average tumor weights were calculated after euthanasia and tumor harvesting. Western blot analysis (**f**) and grayscale scanning (**g**) for detecting G6PD, pSTAT3, and p65 levels in the whole-cell lysates of two random tumor samples. GAPDH levels served as loading controls. Each bar represents the mean ± SD. *P < 0.05, **P < 0.01, ***P < 0.001 vs control or vehicle

The aforementioned results showed that NF-κB and pSTAT3 signaling pathways were likely to drive G6PD overexpression through the constitutively activated effect of p65 and pSTAT3, indicating that G6PD, as an important common mediator of NF-κB and pSTAT3 signaling, might become a potential therapeutic target for ccRCC treatment. Therefore, it was necessary to investigate whether G6PD activity inhibitors had an anti-tumor effect both in vitro and in vivo. The G6PD competitive activity inhibitor 6-aminonicotinamide (6-AN), a nicotinamide analog [43], was first tested in 786-O cells, one of the most highly cited cell lines that exactly clustered with ccRCC and commonly used for xenografts studies [39]. The results demonstrated that the G6PD activity was significantly decreased in a dose-depcendent manner in 786-O cells following treatment with 6-AN (Fig. 6a). Moreover, the anti-proliferative effect was seen in 786-O cells after treated with 6-AN at the indicated time points and doses (Fig. 6b). In addition, the 786-O parental tumor-bearing nude mice were treated intratumor with vehicle or 6-AN to evaluate the tumor-inhibitory activity of 6-AN in vivo. As presented in Fig. 6c–e, both the tumor volume (Fig. 6c, d) and tumor weight (Fig. 6e) were reduced following 6-AN treatment compared with the vehicle. Subsequently, the expression of G6PD, p-STAT3, and p65 in tumor xenografts was compared by Western blot analysis. The results showed that the expression levels of these factors were decreased in the 6-AN-treated tumor compared with the vehicle (Fig. 6f, g). Taken together, these in vivo data confirmed that there may be a positive correlation between G6PD overexpression and NF-κB/STAT3 signaling pathway over-activation. Moreover, G6PD inhibition exhibited tumor-suppressing activities in ccRCC, indicating that G6PD might be a potential therapeutic target for ccRCC treatment.

## Discussion

Increasing evidences including results from our laboratory demonstrated that aberrant expression and activation of G6PD promoted cell proliferation and adaptation in a serial of cancers and might serve as a potential anticancer target [16, 17, 19]. In these studies, the oncogenic functions and underlying molecular mechanisms of G6PD in cancer progression have partially been clarified. However, the question why G6PD is over-activated in different types of cancers is largely unknown.

G6PD activation could be regulated at both transcriptional and post-translational levels. It has been reported that Nrf2 could contribute to elevated G6PD expression in hepatocellular carcinoma through the transcriptional regulatory effect [44]. Ma et al*.* reported that Plk1 interacted with G6PD, promoted the formation of G6PD active homodimer, and therefore promoted cancer cell growth [45]. Meanwhile, Jiang et al*.* suggested that p53 formed a complex with G6PD, but inhibited its activities by restraining G6PD homodimer formation [46]. On the contrary, TAp73, a structural homolog of p53, could activate G6PD transcription, and facilitate oncogenic cell proliferation [47]. Although G6PD could be promoted by the positive feedback regulatory action of pSTAT3 through transcriptional regulation [19], the reason for ectopic G6PD gene overexpression in ccRCC still needs to be unraveled.

**Fig. 7:**
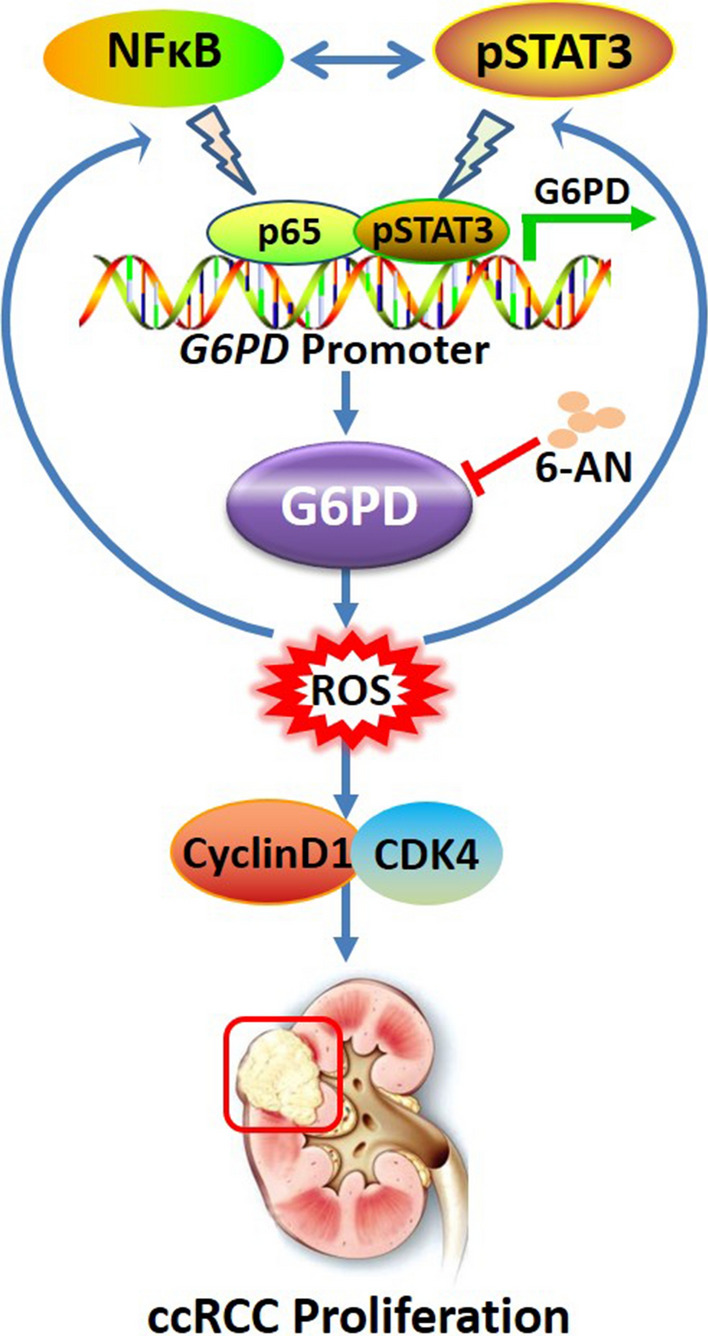
NF-κB and pSTAT3 synergistically drove G6PD overexpression and facilitated sensitivity to G6PD inhibition in ccRCC. ROS-stimulated NF-κB and pSTAT3 signaling over-activation could activate each other, and perform a cross-talk in the process of G6PD transcriptional regulation. The underlying mechanism was that p65 and pSTAT3 formed a p65/pSTAT3 complex, occupied the pSTAT3-binding site on the G6PD promoter, synergistically facilitated G6PD overexpression, and contributed to ccRCC proliferation. Moreover, G6PD activity inhibition might be a potential therapeutic strategy for ccRCC treatment

Previous studies from our laboratory demonstrated that G6PD overexpression lead to the increment of G6PD-NADPH-NOX4-dependent ROS accumulation and then pSTAT3 and MAKP signaling over-activation in ccRCC [19, 20]. However, several problems, such as alterations in ROS-triggered signaling pathways or other relevant regulators responsible for G6PD high expression, remained to be clarified. This study unraveled the interactive signaling pathways involved in G6PD overexpression, including ROS, NF-κB, and pSTAT3. Each of these pathways has been well studied in the tumorigenesis and development of a series of human cancers [22, 29, 33, 48]. ROS production and clearance are closely related to intracellular redox homeostasis and can be regulated by many factors. Compared with normal cells, tumor cells, especially derived from kidney cancers, are in a strong state of oxidative stress [49]. It is noteworthy that G6PD plays an important role in maintaining ROS homeostasis by balancing NADP/NADPH, GSH/GSSG, and other redox systems [16, 17]. Preliminary study showed that not only G6PD regulated ROS production and pSTAT3 activation, but ROS accumulation also impacted G6PD expression [19]. Therefore, it was hypothesized that ROS-involved signaling pathways might participate in G6PD dysregulation, perform cross-talk, and form a feedback loop to promote ccRCC tumorigenesis. The present study demonstrated that significantly reduced or increased activities of the NF-κB signaling pathway was found in ccRCC cells following treatment with ROS scavenger or stimulator, respectively (Fig. 1), which was consistent with the changes in pSTAT3 signaling activity and G6PD expression. The aforementioned evidences suggested that pSTAT3 and NF-κB signaling were transcriptional regulators and might play synergistic effects on G6PD overexpression in ccRCC. After comprehensive investigation, it was concluded that ROS induced NF-κB and pSTAT3 signaling over-activation. These two signaling pathways not only activated each other, but also formed a p65/pSTAT3 transcriptional complex and occupy pSTAT3- but not the NF-κB-binding site while displaying synergistic effects on promoting G6PD transcription (Fig. 7).

This binding site specificity could be caused by many reasons. One of them might be the presence of some other transcriptional cofactor that interacted with p65 or pSTAT3 and facilitated the p65/pSTAT3 complex to occupy the pSTAT3- rather than the NF-κB-binding site in mediating G6PD transcription. This potential cofactor must have the ability to interact with p65 or pSTAT3. Meanwhile, it should be potential to bind to G6PD promoter region which is nearby the pSTAT3- binding site. Here, we propose HIF1α as one of the candidates, which has been reported to be overexpressed and play significant roles in ccRCC tumorigenesis [50]. HIF1α could cooperatively formed a complex with p65 [51] or pSTAT3 [52] to regulate different genes transcription. Furthermore, MatInspector bioinformatic analysis showed that there are two extremely adjacent HIF1α binding sites which were located before and after the pSTAT3-binding site, but relatively far away from the NF-κB-binding site on the transcriptional regulatory region of G6PD. However, whether the interaction between p65 and pSTAT3 is a direct protein–protein effect, or bridged by some other cofactors are need to be further unraveled. Additionally, the other way round, another speculation leading to the binding site specificity of p65/pSTAT3 complex in ccRCC may be the occupation of NF-κB-binding site binding sits by other transcriptional cofactor that caused the existence of DNA helical steric hindrance effects and prevented the binding of p65/pSTAT3 complex on this region. However, further investigations are waiting to be conducted for testifying this hypothesis.

As the first and rate-limiting enzyme of PPP, G6PD not only mediates glucose catabolism and maintains cell redox homeostasis, but also generates sufficient precursors and plays crucial roles in the biosynthesis of lipids and nucleic acids to meet the requirement of cancer cell for rapid proliferation and progression [17, 46]. The present study suggested novel proliferation strategies of ccRCC cancer cells via dual oncogenic transcriptional factors, NF-κB and STAT3, which are over-activated in ccRCC and cooperatively facilitate ccRCC proliferation through inducing G6PD overexpression and then cell cycle regulators such as CyclinD1 and CDK4. Additionally, previous results described that G6PD high expression was positively correlated to lymph node metastasis, Fuhrman grade, and TNM stage of ccRCC, indicating that G6PD might be involved in promoting ccRCC metastasis. Although the mechanism of G6PD in ccRCC was not fully revealed, it was faithful that in addition to the proliferation-promoting effect, the work model unraveled in present study—NF-κB and pSTAT3 synergistically drove G6PD overexpression (Fig. 7)—might display more functions, especially the potential to mediate tumor metabolic reprogramming and facilitate metastasis of ccRCC patients. Therefore, more investigations are necessary to be carried out for clarifying these hypotheses.

Normally, NF-κB is sequestered in the cytoplasm in an inactive form, and bound to one of many inhibitory molecules (IκBs), of which IκBα is the most abundant one. IκBα could form inactive complexes with p50 or p65 in cytoplasm. The phosphorylation of IκBα leads to the active NF-κB being translocated to the nucleus where it binds to the target genes promoter and induces the transcription of oncogenes that regulate almost all important aspects of RCC progression, including proliferation, apoptosis, metastasis and chemotherapy resistance [41]. It is well known that the most studied activated form of NF-κB signaling pathway is the p65/p50 heterodimer, which is stimulated through the canonical pathway and usually promotes gene transcriptional regulation. However, in the present study, we found that pIκBα was reduced by ROS depletion, and NF-κB signaling pathway facilitated G6PD expression via direct protein–DNA interaction with only p65 but not p50 binding to the NF-κB motif at G6PD promoter. Chromatin binding efficiency of the p50 and p65 antibodies used in this study was validated according to some published reports [53, 54], and the results demonstrated that both these antibodies worked in ChIP assays. Many studies have suggested that p65 is different from p50 in regulating the transcription of targeted genes [55–57]. For instance, it is the p65/p65 or p50/p50 homodimer, not the canonical p65/p50 heterodimer, which directly binds to the promoter and regulates the target genes expression to mediate cell physiological functions [54, 56, 58]. These observations support the idea that p65 and p50 might display different characteristics in interacting with the potential NF-κB binding site, and p65 which is highly expressed in ccRCC tumor tissues might exert more important roles in promoting G6PD transcription in ccRCC. Moreover, disproportionate increase in activated p65 and subsequent transactivation of effector molecules have been reported to be involved in the pathogenesis of multiple diseases including the rheumatoid arthritis, inflammatory bowel disease, colon carcinoma, and even neurodegenerative pathologies [54, 59]. Our present results also demonstrated that ROS activated NF-κB could form a p65/pSTAT3 transcriptional complex and facilitate G6PD transcription, which might be due to the heterogeneity of ccRCC or unbalanced proportion of p65 and p50 under ROS stimulation in ccRCC. The above reports and our intriguing findings indicated that the NF-κB p65 signaling pathway might become a pivotal target for potential drug discovery and development in the inflammation and tumor-related treatment [59].

In addition, when the endogenous expression of p65 was knocked down by RNAi lentivirus, not only p65 levels were declined, a significant reduction of p105 and p50 was also found in ccRCC cell lines, and vice versa (Fig. 2f–g). These results indicated an interaction between p65 and p50—two functional activators of NF-κB signaling pathway. Additionally, p50 also responded to ROS stimulation (Fig. 1c, d) and could form a complex with pSTAT3 (data not shown). However, the G6PD protein expression levels were not significantly modified by p50 knocked down, and p50 recruited on neither the potential NF-κB-binding site (Fig. 2e) nor the pSTAT3-binding site (Fig. 3b) on the G6PD promoter, suggesting that p50 may be potential to interact with pSTAT3, but play no roles in promoting G6PD overexpression, and this complex might occupy other undiscovered binding sites. Therefore, more experiments to verify the impact of p50 on G6PD expression and the interaction between p65, p50, and pSTAT3 in ccRCC still need to be performed in future studies.

Moreover, G6PD has been pinpointed as a new biomarker in acute myeloid leukemia (AML), and its overexpression positively correlated with poor prognosis of AML patients [60]. Meanwhile, targeting G6PD induces apoptosis and enhances chemotherapeutic antitumor effects via ROS-mediated damage in certain cancer, including AML, lung cancer, breast cancer, and colorectal cancer [60, 61]. Previous study also indicated that G6PD was a potential prognostic biomarker and a promising therapeutic target for ccRCC treatment [19]. However, whether G6PD inhibition exert any antitumor effects in ccRCC is far from being clarified. The present study investigated whether the nicotinamide analog 6-AN could affect the proliferation of ccRCC both in vitro and in vivo. 6-AN, a known competitive inhibitor of G6PD, could modulate the cytotoxicity of antineoplastic treatments, and is undergoing preclinical investigation in certain cancers [43, 62]. As shown in Fig. 6a, b, a significantly reduced proliferation rate of 786-O cells was found in vitro. However, the inhibition of tumor growth was not as dramatic as that mediated by G6PD silencing in our previous study [19], which most probably due to low drug bioavailability. Nevertheless, the inhibition of tumor growth was associated with decreased levels of G6PD, pSTAT3, and p65 in isolated tumor samples (Fig. 6f, g), demonstrating that abnormally activated pSTAT3 and NF-κB signaling pathways were positively correlated to G6PD overexpression in vivo and G6PD inhibition exhibited tumor-suppressive activities in ccRCC. Although developing clinical application are still challenging, the present studies indicated that G6PD-based gene therapy might provide an adjunctive approach to ccRCC treatment.

## Conclusion

Collectively, this study aimed to unravel the reason for abnormal G6PD overexpression and further revealed the proliferation repressive effect of G6PD inhibition in ccRCC. The results demonstrated ROS-stimulated NF-κB and pSTAT3 signaling over-activation could activate each other, and perform a cross-talk in the process of G6PD transcriptional regulation. The underlying mechanism was that p65 and pSTAT3 formed a p65/pSTAT3 complex, occupied the pSTAT3-binding site on the G6PD promoter, synergistically facilitated G6PD overexpression, and contributed to ccRCC proliferation. Moreover, G6PD activity inhibition might be a potential therapeutic strategy for ccRCC treatment (Fig. 7). Despite the aforementioned work model, the question why G6PD exhibits aberrant activities in other types of human cancers is far from being answered. Meanwhile, G6PD is a crucial metabolic regulatory factor. It is tentatively to hypothesize that G6PD must participate in the entire metabolic reprogramming of ccRCC. Overall, the results shed new light on the mechanism underlying G6PD dysregulation. Other functions and relevant mechanisms of G6PD in ccRCC carcinogenesis and metabolic reprogramming should be explored in future study.

## Data Availability

The data used to support the findings of this study are available from the corresponding author upon request.
