# Correction to: NF-κB and pSTAT3 synergistically drive G6PD overexpression and facilitate sensitivity to G6PD inhibition in ccRCC

**DOI:** 10.1186/s12935-020-01664-3

**Published:** 2020-11-26

**Authors:** Qiao Zhang, Zhe Yang, Yueli Ni, Honggang Bai, Qiaoqiao Han, Zihan Yi, Xiaojia Yi, Yannick Luther Agbana, Yingmin Kuang, Yuechun Zhu

**Affiliations:** 1 Department of Biochemistry and Molecular Biology, School of Basic Medical Sciences, Kunming Medical University, No. 1168 Yuhua Road, Chenggong District, Kunming, 650500 Yunnan China; 2 Department of Pathology, The First Affiliated Hospital of Kunming Medical University, No. 295 Xichang Road, Wuhua District, Kunming, 650032 Yunnan China; 3 Department of Clinical Laboratory, The Second Hospital of Jingzhou, No. 241 Jiangjin Road, Shashi District, Jingzhou, 434000 Hubei China; 4 Department of Medical Oncology, The Third Affiliated Hospital of Kunming Medical University (Tumor Hospital of Yunnans Province), No. 519 Kunzhou Road, Xishan District, Kunming, 650118 Yunnan China; 5 Department of Pathology, The Second Affiliated Hospital of Kunming Medical University, No. 374 Dianmian Road, Wuhua District, Kunming, 650101 Yunnan China; 6 Department of Organ Transplantation, The First Affiliated Hospital of Kunming Medical University, No. 295 Xichang Road, Wuhua District, Kunming, 650032 Yunnan China

## Correction to: Cancer Cell Int 20:483 (2020) 10.1186/s12935-020-01576-2

After publication of the original article [1], the authors have notified us that the affiliation of author Yuechu Zhu should read “1” instead of “2”.

Correct author affiliation:

Department of Biochemistry and Molecular Biology, School of Basic Medical Sciences, Kunming Medical University, No. 1168 Yuhua Road, Chenggong District, Kunming 650500, Yunnan, China.
